# Purification and Biochemical Characterization of *Sucrose synthase* from the Stem of Nettle (*Urtica dioica* L.)

**DOI:** 10.3390/ijms22020851

**Published:** 2021-01-16

**Authors:** Lavinia Mareri, Gea Guerriero, Jean-Francois Hausman, Giampiero Cai

**Affiliations:** 1 Dipartimento Scienze della Vita, Università di Siena, via Mattioli 4, 53100 Siena, Italy; cai@unisi.it; 2 Environmental Research and Innovation (ERIN) Department, Luxembourg Institute of Science and Technology (LIST), 5 rue Bommel, Z.A.E. Robert Steichen, L-4940 Hautcharage, Luxembourg; gea.guerriero@list.lu (G.G.); jean-francois.hausman@list.lu (J.-F.H.)

**Keywords:** *Urtica dioica*, *Sucrose synthase*, bast fibers, protein purification, phosphorylation

## Abstract

*Sucrose synthase* is a key enzyme in sucrose metabolism as it saves an important part of sucrose energy in the uridine-5′-diphosphate glucose (UDP-glucose) molecule. As such it is also involved in the synthesis of fundamental molecules such as *callose* and *cellulose*, the latter being present in all cell walls of plant cells and therefore also in the gelatinous cell walls of sclerenchyma cells such as bast fibers. Given the importance of these cells in plants of economic interest such as hemp, flax and nettle, in this work we have studied the occurrence of *Sucrose synthase* in nettle stems by analyzing its distribution between the cytosol, membranes and cell wall. We have therefore developed a purification protocol that can allow the analysis of various characteristics of the enzyme. In nettle, *Sucrose synthase* is encoded by different genes and each form of the enzyme could be subjected to different post-translational modifications. Therefore, by two-dimensional electrophoresis analysis, we have also traced the phosphorylation profile of *Sucrose synthase* isoforms in the various cell compartments. This information paves the way for further investigation of *Sucrose synthase* in plants such as nettle, which is both economically important, but also difficult to study.

## 1. Introduction

*Sucrose synthase* (SuSy; EC 2.4.1.13) is a key enzyme involved in sucrose metabolism, which catalyzes the reversible transformation of sucrose into fructose and UDP-glucose [1]. This enzyme belongs to the glycosyltransferase-4 subfamily of the carbohydrate-active enzyme (CAZyme) repertoire, a large family that also includes other glycosyltransferases such as trehalose synthase, sucrose phosphate synthase and trehalose phosphorylase [2]. With some exceptions, *Sucrose synthases* are typically homotetramers, with an average weight for each monomer of about 90 kDa, measuring 800 amino acids in length [2]; some SuSy isoforms are considerably different, meaning their weights are accordingly different, as reported for *Arabidopsis thaliana* [3] and banana (*Musa acuminata*) [4], whose monomers are 107 kDa and 110 kDa, respectively. The molecular weight of the SuSy monomer may be even smaller, as reported in studies in bird cherry [5], wheat [6] and azuki bean [7]. Each monomer is composed of an N-terminal domain of about 250 amino acids that is supposed to be involved in cellular targeting and a C-terminal GT-B domain of about 500 amino acids that is responsible for the enzyme’s glycosyltransferase activity [8]; the authors also confirmed the tetrameric structure of plant SuSy using X-ray crystallography. SuSy can have multiple subcellular localizations [1]. Initially, SuSy was supposed to be cytosolic, as reported in bean plants [9], but later evidence showed that it can be also located in the plasma membrane [10,11,12] or in the cell wall compartment [11,13]. The association of SuSy with membranes is supposed to be mediated by the phosphorylation of serine residues at position 11 to 15 in the N-terminal domain; in maize, membrane association is decreased by phosphorylation, while dephosphorylation makes SuSy less soluble [14]. On the contrary, other studies indicated that the N-terminal serine residue might not be essential for the subcellular localization, as reported in studies in soybean [15] and mung bean [16]. Few isoforms of SuSy have been reported in cell walls. For example, in cotton fibers, SusC (namely cell wall-associated SuSy) was reported to be located in cell walls during secondary cell wall synthesis, and may play a role in *cellulose synthesis* [17]; in tobacco pollen tubes, the authors observed cell-wall-associated SuSy [11]. However, the mechanism that regulates the association of SuSy with the cell wall is still unclear and may be due to a truncated N-terminus and C-terminus [17].

SuSy gene (*SUS*) families are generally small, containing between five to eleven genes; an exception is Chinese pear (*Pyrus bretschneideri* Rehd.), where at least 30 different *SUS* genes have been characterized [18]. Plant *SUS* genes are divided into three separate clades, which are present in both monocots and dicots. A comprehensive phylogenetic analysis indicates that a first SUS duplication event may have occurred before the divergence of gymnosperms and angiosperms and a second duplication event probably occurred in a common angiosperm ancestor, leading to the existence of all three clades in both monocots and dicots [1].

SuSy plays important roles in plant sugar metabolism, principally in sink tissues [1]. Sucrose can enter the sink cells via several different pathways, and once inside the cell it can take different paths. In the cytosol, sucrose can be hydrolyzed by cytosolic invertase to yield glucose and fructose or by cytosolic SuSy to yield fructose and UDP-glucose. The synthesized hexoses can, thus, be phosphorylated (hex-P) and directed to starch synthesis in the plastid or to glycolysis in the mitochondria. In this regard, many observations support the involvement of SuSy in starch accumulation. For example, in potato, carrot taproots and maize endosperm, starch content was reported to be decreased as a consequence of SuSy activity reduction [19,20,21]. Plasma-membrane-associated SuSy (pmSuSy) and cell-wall-associated SuSy can generate UDP-glucose, which is a direct substrate for both *cellulose* (β1-4) and *callose* (β1-3) glucans used in the synthesis of *cellulose* and *callose*, the latter used both during developmental stages and in response to biotic or abiotic stress. Many studies highlight the contribution of SuSy to the synthesis of both polysaccharides. Evidence for a role of SuSy in *callose* deposition was found in an *Arabidopsis* double mutant of phloem-specific *SUS* (*sus5 sus6*). The double mutant had less *callose* in its phloem plasmodesmata and in response to leaf wounding as compared with wild-type or quadruple mutant (*sus1*, *sus2*, *sus3* and *sus4*) plants [22]. Involvement of SuSy in *callose* deposition has also been suggested by co-distribution analysis [23].

The role of SuSy in the synthesis of *cellulose* and *callose* has been well investigated in cotton fibers, which are a model for these processes. In the first phase of cotton fiber development, epidermal cells elongate, followed by massive *cellulose* production. In the secondary growth phase, the synthesis of *cellulose* is increased by around 100-fold compared to the elongation phase [24]. In agreement with the involvement of SuSy in the synthesis of *cellulose* and *callose*, Ruan et al. [25] and Ruan [26] showed that transgenic cotton plants with *SUS* suppression exhibit reduced fiber initiation and elongation. Further evidence of the involvement of SuSy in *cellulose* and *callose synthesis* also emerged from data showing an association between SuSy and *cellulose synthase* [7] or *callose synthase* [27].

Plants that produce bast fibers are extremely important because they provide both long and strong fibers containing high amounts of *crystalline cellulose*. These fibers have applications in the textile industry, as well as in the biocomposite industry, being environmentally friendly alternatives to artificial fibers [28]. Flax (*Linum usitatissimum* L.), hemp (*Cannabis sativa* L.) and nettle (*Urtica dioica* L.) are fibrous plants that have attracted more interest as producers of fiber cells with gelatinous cell walls, high crystalline *cellulose* content and low lignin content [29,30].

While flax and hemp have received considerable interest from the scientific community, nettle has not been studied as much and can still be considered an underestimated plant among those of economic interest. In nettle, there is a gradient of lignification and strengthening of the stem that progressively proceeds from the top to the bottom [31]. This reinforcing mechanism is based on the increasing number of bast fibers in the stem and on the thickening of their secondary cell walls. The increase in the content of *cellulose* and other pectic or hemicellulosic components requires a more active metabolism of sugars and their channeling towards the synthesis of polysaccharides. It is, therefore, reasonable to assume that SuSy may be differentially active in top or bottom regions or that differing gene expression may be present. An earlier work identified six *SUS* genes in nettle belonging to three previously reported Angiosperm groups (groups I–III). The gene expression analysis proved a differential regulation in stem samples at different heights corresponding to an increase in cell wall thickness of bast fibers along the nettle stem. Three genes were either more expressed in young or old stem segments or in the middle internode [32]. Analysis of the *SUS* gene expression in nettle has, therefore, shown an expression pattern that follows the developmental profile of bast fibers.

In order to better understand how SuSy participates in the development of the gelatinous cell walls of bast fibers, it is also necessary to analyze the behavior of SuSy from a biochemical point of view; as far as we know, no biochemical studies on SuSy are available for nettle. Therefore, in this work we analyze SuSy in nettle stems using a commercial antibody directed against SuSy-1 from *Arabidopsis*. There are currently no antibodies directed against nettle SuSy and only a small number of antibodies against SuSy are commercially available (and their cross-reactivity against nettle SuSy is not known). We study the distribution of SuSy between the cytosol, membranes and cell wall and design a protocol for the purification of SuSy from the cytosol fraction, as well as to analyze the different phosphorylation pattern of SuSy isoforms in the cytosol, membranes and cell wall.

## 2. Results

### 2.1. Characterization of the Anti-SuSy Antibody

**Figure 1:**
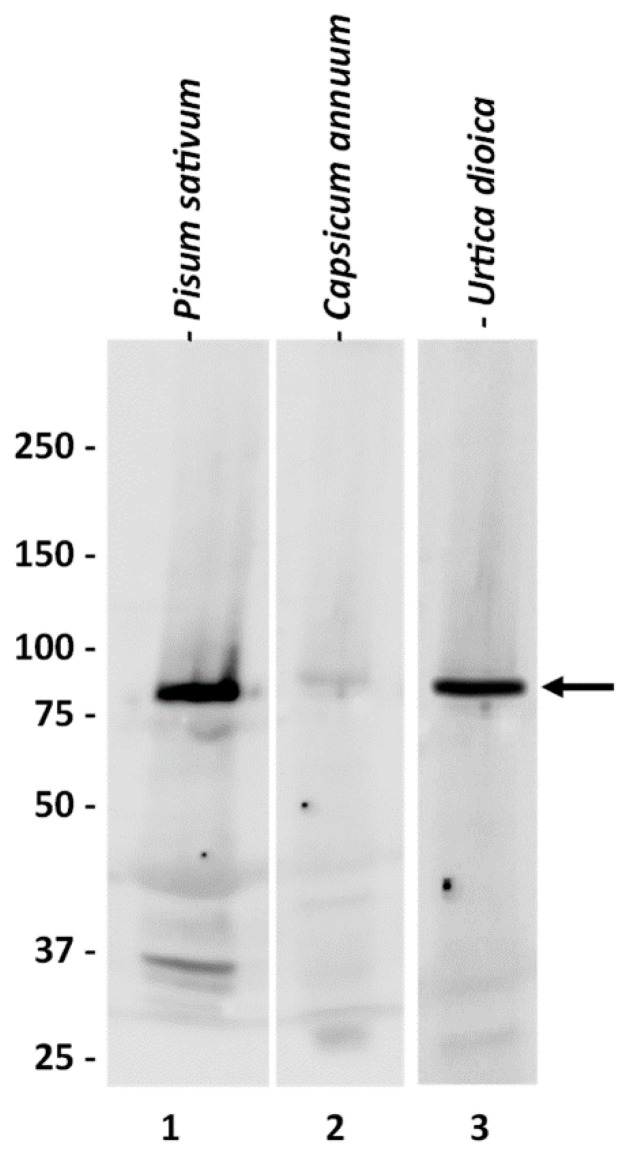
Western blot analysis of the specificity of the commercial antibody against sus-1 of *Arabidopsis thaliana*. Lane 1, extract from *Pisum sativum* leaves; lane 2, protein extract from *Capsicum annuum* leaves; lane 3, protein extract from *Urtica dioica* leaves. The three lanes contained about 30 μg of protein. The anti-sus-1 antibody highlights a band with a molecular weight slightly higher than 75 kD in all three samples, although less intensely in the *Capsicum annuum* protein extract. Numbers on the left indicate the molecular weights in kD. The arrow indicates the position of the cross-reactive band.

As a first step, we characterized the commercially obtained anti-SuSy antibody. The choice of this specific antibody was largely dictated by the limited availability of anti-SuSy antibodies on the market. On the other hand, the choice was also affected by the evidence that this antibody has a confirmed reactivity against the Sus-1 isoform of *Arabidopsis*, which in turn shows a good sequence homology with the nettle *SUS* gene family. Particularly, the BLASTp analysis showed that UdSUS1 has the highest percentage of identity with the corresponding *Arabidopsis* sequence (86.46%), while the homology between the other UdSUS proteins was lower, ranging between 81% for UdSUS3 and 71% for UdSUS6. As detailed later in the text, some of the nettle sequences are incomplete. The lower score is a consequence of the lack of full-length sequences for some of the nettle SuSy genes. We then tested the anti-SuSy antibody on two plant extracts (*P. sativum*, *C. annuum*), as well as on nettle (Figure 1). In the three plants tested, the antibody showed cross-reactivity against a specific molecular weight band above 75 kDa. The signal was very specific, and the molecular weight was as expected. The signal was very intense in the case of *Pisum* (for which the company reported a predicted reactivity) and *Urtica*, and much less so (but still specific) in the case of *Capsicum*. Although we did not have the sequence of the protein identified by the antibody, the high specificity and molecular weight value gave us confidence in the quality of the signal obtained. The antibody was then used as a probe to trace the nettle SuSy during the purification process.

### 2.2. Purification of Cytosolic SuSy from Nettle Stems

The purification of SuSy from nettle stems was carried out on the basis of existing protocols. The enzyme has been purified from distinct species, tissues and organs. We, therefore, designed a protocol that considered the already published methods, as well as personal experience. In the end, we preferred a simplified protocol based on anion exchange and gel filtration chromatography. Alternatively, we also used hydrophobic interaction chromatography in the third step, however the output was too low.

**Figure 2:**
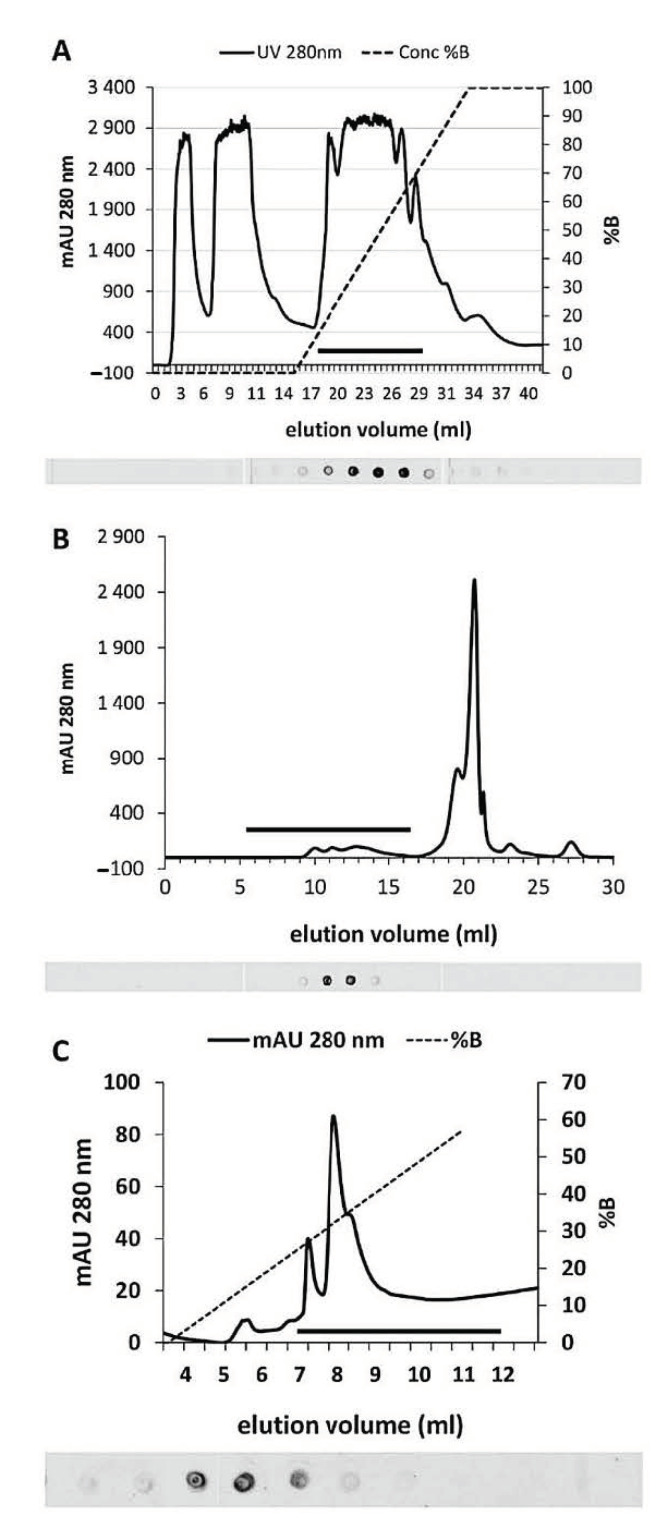
Stages of purification of *Sucrose synthase* from nettle stems. (**A**) Graph of ion-exchange chromatography on a Resource-Q or HiTrap-Q column. The absorbance profile at 280 nm and the percentage profile of the NaCl gradient (dotted line) used to elute proteins are shown. The black bar at the bottom indicates the fractions whose spot test is shown just below. Spot test analysis highlights the position of *Sucrose synthase*. (**B**) Elution profile by gel filtration chromatography used as the second step of purification of *Sucrose synthase*. The graph shows the absorbance at 280 nm. The horizontal bar also corresponds to the spot test shown just below, in which the fractions enriched in *Sucrose synthase* are highlighted. (**C**) Graph of the third step of purification of *Sucrose synthase* by anion exchange chromatography on a Mini-Q column. The 280 nm absorbance profile and the percentage profile of the NaCl gradient (dotted line) used to elute the proteins are shown. The horizontal black bar at the bottom corresponds to the spot test shown just below where the fractions containing *Sucrose synthase* are highlighted.

The first purification step was chromatography on an anion exchange column (Resource Q instrument, however we also used a Hi-Trap Q column and achieved equivalent results). As shown in the graph of Figure 2A, SuSy was localized in a small number of fractions corresponding to elution volumes ranging between 23 and 26 mL. The graph in Figure 2A shows the absorbance profile at 280 nm and the increasing concentration of NaCl used in the chromatography. Below this, a part of the spot test used to locate SuSy is highlighted. The black bar in the graph indicates the area corresponding to the fractions analyzed by spot tests. The positive fractions were then subjected to gel filtration chromatography, the results of which are shown in Figure 2B. Even in this case, the black bar in the graph corresponds to the spot test shown just below. SuSy was located in a fairly small number of fractions, roughly corresponding to an elution volume of ca. 12 mL. When compared to standard proteins, this position returns a molecular weight of about 300 kDa. This value suggests that SuSy is not monomeric and could exist in a tetrameric form. Positive fractions of the gel filtration were then further analyzed by anion exchange chromatography on a Mini-Q column, which is a high-resolution column. The results are shown in Figure 2C. The black bar in the graph corresponds to the fractions analyzed in the spot test below. SuSy elutes in a small number of fractions corresponding to 8–9 mL of the elution volume. Since some protocols in the literature use hydrophobic interaction chromatography for the purification of SuSy, the third step of our protocol was sometimes replaced by hydrophobic interaction chromatography using a Resource-Phe column. Unfortunately, the results were not satisfactory, and therefore such attempts were abandoned.

**Figure 3:**
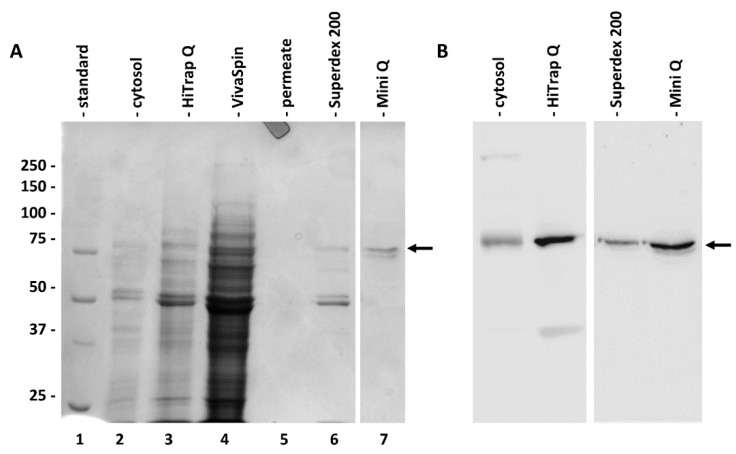
Electrophoretic summary of the purification of *Sucrose synthase*. (**A**) Gels electrophoresis of the main fractions obtained during purification. Lane 1, molecular weight standard whose value in kD is shown on the left; lane 2, cytosol fraction; lane 3, pool after anion exchange chromatography with a HiTrap-Q column; lane 4, the same sample after concentration with a VivaSpin; lane 5, the permeate; lane 6, pool after gel filtration chromatography with a Superdex 200 column; lane 7, pool after anion exchange chromatography with a Mini-Q column. The arrow on the right indicates the position of the purified band. (**B**) Western blot analysis with the anti-sus-1 antibody on some fractions shown in A, particularly the cytosol fraction, as well as the pool after HiTrap-Q and the pools after Superdex 200 and Mini-Q chromatography. The arrow on the right indicates the position of the cross-reactive band.

The main fractions obtained during the chromatography purification steps were then analyzed individually by electrophoresis and immunoblotting (Figure 3). Figure 3A shows the electrophoretic profile of the cytosol (lane 2), anion exchange chromatography (in this case performed with a HiTrap-Q column, lane 3) of the same sample concentrated by VivaSpin (lane 4; as well as the corresponding permeate, lane 5) and then of the pool obtained after gel filtration chromatography with a Superdex 200 column (lane 6) and after the last step of ion-exchange chromatography with a Mini-Q column (lane 7). As indicated by the arrow on the right, at the end of the purification process, a band with a molecular weight above 75 kDa was obtained; a further faint band of lower molecular weight was sometimes present. The corresponding immunoblotting of Figure 3B confirms the progressive purification of SuSy. The antibody signal is evident (even if not very strong) in the cytosol fraction; on the contrary, it is of good intensity in both the fraction after HiTrap-Q chromatography and after subsequent chromatography with the Superdex 200 column and Mini-Q column.

### 2.3. Sequential Extraction of SuSy from the Cytosol, Membranes and Cell Wall

**Figure 4:**
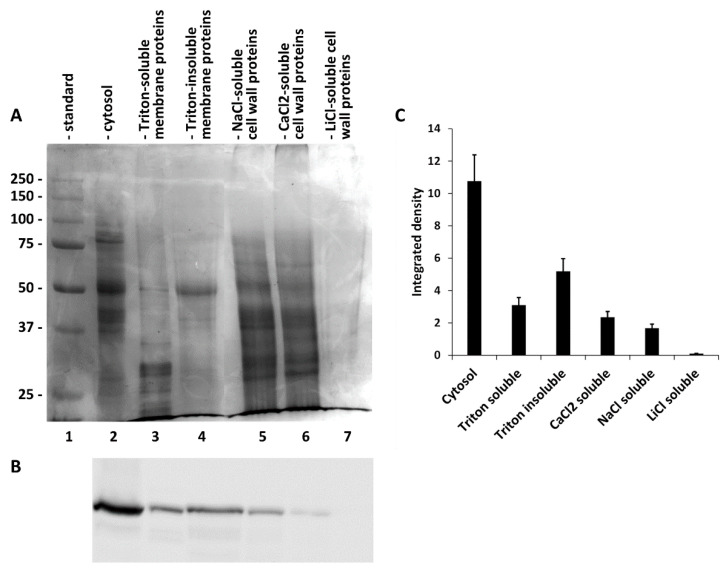
(**A**) Gel electrophoresis assessment of protein fractions obtained using differential extraction from nettle stems. Lane 1, molecular weight standard, whose value in kD is shown on the left; lane 2, cytosol proteins; lane 3, Triton-soluble membrane proteins; lane 4, Triton-insoluble membrane proteins; lane 5, cell wall proteins extracted with NaCl; lane 6, cell wall proteins extracted with CaCl_2_; 7, cell wall proteins extracted with LiCl. All lanes except for number 7 contained the same amount of protein. (**B**) Western blot analysis of the same fractions presented in A with the antibody against *Arabidopsis*-1. (**C**) Quantization of western blot presented in (**B**) using ImageJ software. The intensity values of the bands are reported as the integrated density on the Y-axis.

Since SuSy is a protein that is present in different intracellular compartments, we thought it important to determine its presence in different fractions, including the cytosol, membranes and cell wall. To this end, we fractionated a raw extract of nettle stems using protocols that had already been published in the literature and used for similar purposes in distinct species. At the end we obtained a fraction of the cytosol (Figure 4A, lane 2), a fraction of proteins extracted from membranes by Triton X-100 (lane 3), the corresponding fraction of proteins not extracted from membranes by Triton (lane 4) and subfractions of proteins extracted from the cell wall under different conditions using NaCl (lane 5), CaCl_2_ (lane 6) and LiCl (lane 7). As shown in Figure 4A, all fractions contained proteins, with the exception of the protein fraction extracted from the cell wall by LiCl. The protein profiles of the various fractions were quite different, suggesting that the differential extraction had worked. On the same fractions we then analyzed the presence of SuSy using immunoblotting (Figure 4B). As can be seen, the protein was present in all of the fractions obtained, except in the fraction of proteins extracted from the cell wall by LiCl. With the same protein load, the levels of SuSy were quite different in the various samples, with cytosol being the most abundant fraction in SuSy. A quantization of immunoblotting (Figure 4C) highlighted how the cytosol had a quantity of SuSy three times higher than that present in the fraction of Triton-solubilized membrane proteins and about twice as much as the fraction of Triton-insolubilized membrane proteins. The levels of SuSy in the fractions of proteins extracted from the cell wall were quite low.

### 2.4. Bioinformatics Analysis of Putative Phosphorylation Sites of SuSy

**Figure 5:**
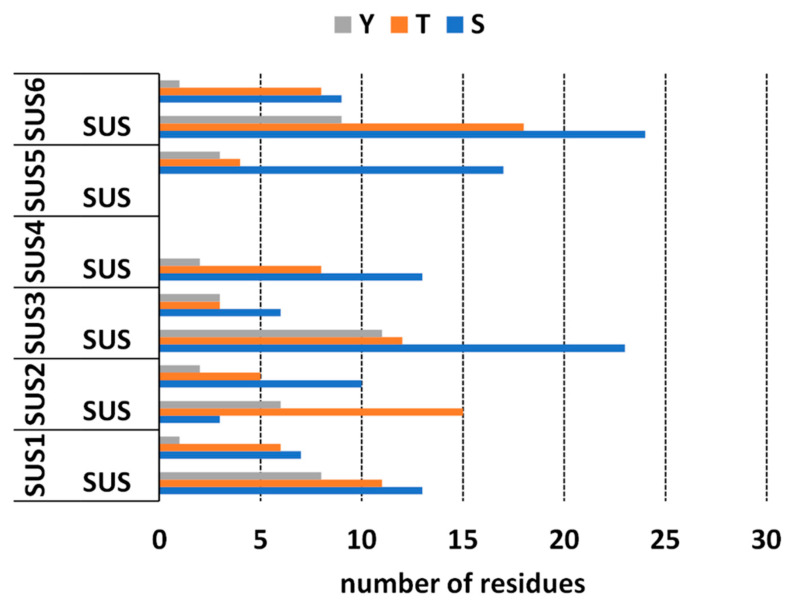
The predicted phosphorylation sites of UdSUS proteins. SUS = *Sucrose synthase* domain; Y = tyrosine; T = threonine; S = serine.

SuSy is a protein that is phosphorylated in several amino acid residues. Based on the results in the literature, the phosphorylation status of the protein can affect its distribution and its enzyme activity. Since we had determined the presence of SuSy in several cellular subfractions, we considered it appropriate to determine the phosphorylation pattern of the various isoforms of nettle SuSy using bioinformatics analysis. In silico analysis of the phosphorylation pattern of UdSUS proteins was performed using the NetPhos 3.1 server [33,34] (Figure 5). Unfortunately, for UdSUS4 and UdSUS5 it was not possible to obtain a global overview of the phosphorylation pattern because the corresponding protein sequences were partial, lacking the SuSy and glycosyl transferase domain, respectively. Results showed that UdSUS1, UdSUS3, UdSUS4 and UdSUS6 in the SuSy domain serine (S) were more phosphorylated than threonine (T), followed by tyrosine (Y), while UdSUS2 had a prevalence of phosphorylated T; as for the glycosyl transferase domain, the trend for all the isoforms was similar, with S sites being more phosphorylated than T and Y and Y being markedly lower than S and T. As for the kinases responsible for the phosphorylation sites, for all UdSUS proteins the most represented were PKA, PKC and CKII, which were similarly distributed along both domains. Non-specific prediction of kinase activity was also detected.

### 2.5. Analysis of SuSy Phosphorylation after Two-Dimensional Electrophoresis

**Figure 6:**
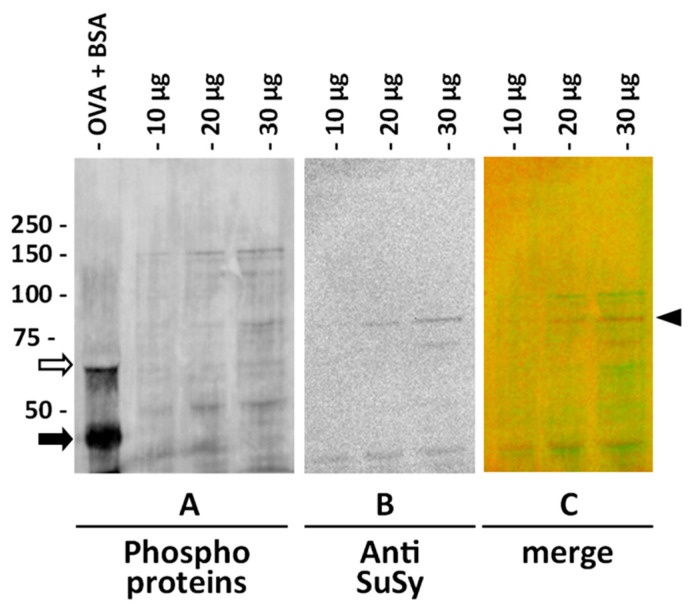
Test with the probe for phosphoprotein and immunoblotting with anti-sus-1 antibody on the cytosolic extract of nettle stem. (**A**) PVDF (polyvinylidene difluoride) membrane stained with phosphoprotein probe. The first lanes contained two reference proteins, BSA (white arrow, molecular weight of about 66 kD), which is not phosphorylated, and chicken ovalbumin (black arrow, molecular weight of about 45 kD), which is phosphorylated. The other lane contained increasing amounts of cytosol protein. (**B**) The same membrane in (**A**) tested with the antibody to sus-1 of *Arabidopsis*. SuSy is detectable as a band at about 80 kD. (**C**) Merging of the two previous images; the phosphoproteins are shown in green, while the signal of SuSy is shown in red (arrowhead).

For the analysis of SuSy phosphorylation, we used a probe directed against phosphorylated amino acids produced by ThermoFisher. According to the manufacturer’s instructions, the probe does not discriminate between different phosphorylated amino acids, and therefore provides an overview of the phosphorylation status of proteins. As a first step, we evaluated the probe using one-dimensional immunoblotting on cytosolic extracts of nettle stem. Two reference proteins, Bovine Serum Albumin—BSA (as non-phosphorylated protein) and chicken ovalbumin (as phosphorylated protein), were run together with the nettle extract (Figure 6). Three different amounts of cytosolic extract were then analyzed with the phosphoprotein probe, thereby providing the image in Figure 6A. After staining for phosphoproteins, the same membrane was then treated for antibody labeling according to the standard protocol, thereby obtaining the image in Figure 6B, where the band recognized by the anti-sus-1 antibody can be seen (in addition to some bands of lower molecular weight). The image in Figure 6C is the merge in pseudo colors of the two previous images; the phosphoproteins are shown in green, while the signal of SuSy is shown in red, indicated by the right arrowhead.

**Figure 7:**
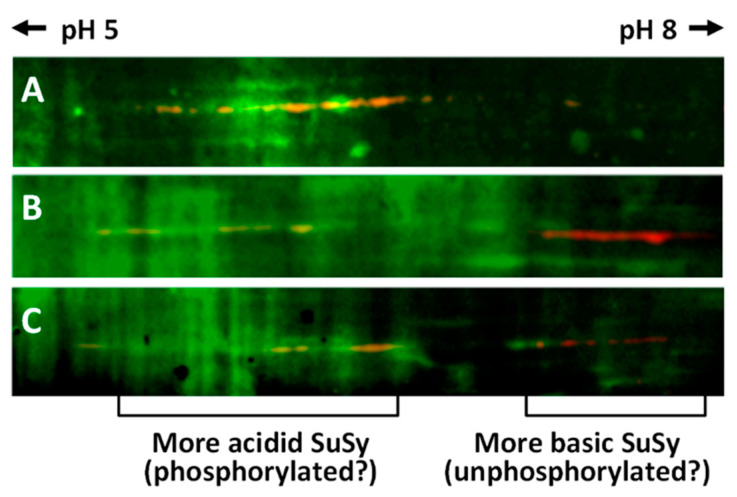
Phosphoprotein staining and western blotting with anti-sus-1 antibody after separation of proteins by two-dimensional electrophoresis with a pH gradient of 5–8. (**A**) Cytosol proteins. (**B**) Membrane proteins. (**C**) Cell wall proteins. The images are merged from the two analyses. The phosphoproteins are shown in green, while the SuSy signal is shown in red. Where the red color turns to orange-yellow, this represents alignment of phosphoproteins and SuSy.

In order to gain more information about the phosphorylated isoforms of SuSy in nettle stems, proteins from the three main cell compartments (cytosol, membranes and cell wall) were subjected to analysis by two-dimensional electrophoresis on strips with pH values of 5–8. Following the second dimension run and transfer to the PVDF membrane, proteins were stained with the phosphoprotein probe and PVDF membranes were then processed for labeling with the anti-SuSy antibody. On completion of the procedure and merging of the resulting images, we obtained the visualization shown in Figure 7. In Figure 7A (cytosol), the region of SuSy accumulation can be seen as a series of yellow-orange spots shifted towards the most acidic region of the pH gradient. In the most basic region, on the other hand, no signal was observed. Figure 7B shows the distribution of SuSy isoforms in protein samples extracted from cell membranes. In this case, two distinct regions of SuSy accumulation can be noted, one more acidic towards pH 5 (whose yellow-orange color indicates overlapping with phosphorylated spots), and one more basic towards pH 8, characterized by red spots (i.e., an absence of phosphorylated amino acids). The proteins extracted from the cell wall (Figure 7C) provided a pattern similar to that from the cell membranes; a more acidic region with yellow-orange SuSy spots (indicating phosphorylated amino acids) and a more basic region with red spots (i.e., no phosphorylation) can be distinguished. Ultimately, two-dimensional electrophoresis allowed us to separate the nettle SuSy into two main clusters, one more acidic and phosphorylated, and one more basic but not phosphorylated. Cytosolic SuSy is characterized only by more acidic and phosphorylated isoforms.

### 2.6. Two-Dimensional Characterization of SuSy Isoforms

**Figure 8:**
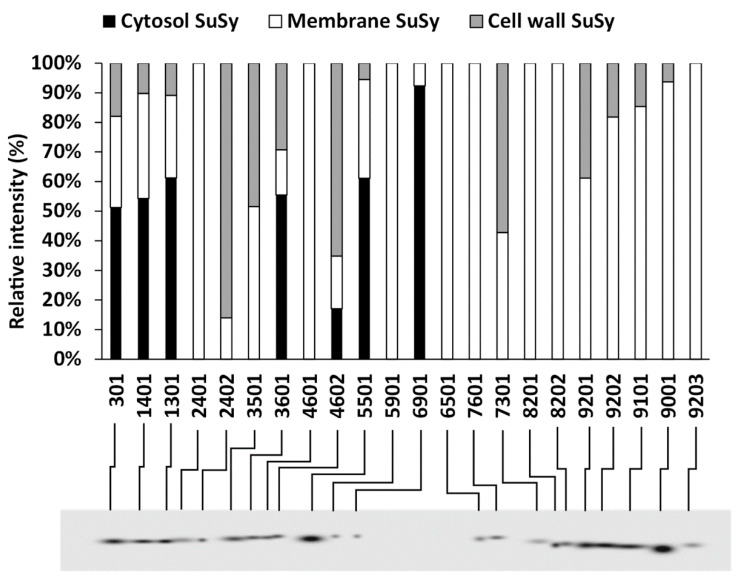
Alignment of SuSy spots obtained from the analysis of the two-dimensional electrophoresis of proteins of cytosol (black bars), membranes (white bars) and the cell wall (grey bars) of nettle stem samples. The intensity of spots is defined as the percentage of relative intensity. Spot numbering was done automatically using PDQuest software. Below, the virtual blot (master blot) summarizes all of the SuSy spots identified in the three samples.

The results of the two-dimensional electrophoresis assessment were then compared with respect to the spot position. This choice was made because of the similarity of the SuSy patterns in the comparison between the cytosol, cell membranes and cell wall. For this purpose, we used PDQuest software, which allowed us to align the various spots that had been identified. The graph in Figure 8 shows the relative percentage of individual spots classified in the analyzed fractions. The identification number for each spot is automatically determined by the software. The blot below represents a so-called “master blot”, i.e., a virtual blot including all of the SuSy spots identified in the three fractions. The graph indicates that some spots (those represented by black bars) are much more abundant in the cytosol and that all of these spots fall into the acid region of blots. The basic region is characterized by the absence of cytosolic SuSy and by the presence mainly of SuSy associated with cell membranes. Some SuSy spots of the cell wall also fall into the basic region, which is, however, much more enriched in cell membrane SuSy. Interestingly, the acid region contains some isoforms that are more representative of the cell wall. None of the spots are found equally in the three fractions. In conclusion, the spot comparison indicates that the acidic region is mainly characterized by isoforms belonging to SuSy of the cytosol and also of the cell wall, while the basic region is, on the contrary, more characterized by isoforms belonging to membrane SuSy.

## 3. Discussion

In this manuscript, we have analyzed some of the biochemical characteristics of SuSy from nettle stem samples. The reason to focus on *U. dioica* is motivated by the multi-purpose applications of this weed and the still neglected potential it has to serve a bioeconomy. It is undeniable that other multi-purpose crops, such as *Cannabis sativa* or *Linum usitatissimum*, have so far attracted much more attention [35,36]; however, to reach a phase whereby the dependence of industry on petrochemicals is greatly reduced, it is important to diversify the sources of biorenewable raw materials. Nettle can fulfil such requests, since it grows rapidly, is robust and is a phytofactory producing long and strong bast fibers. More specifically, *U. dioica* is used as a source of phytochemicals with relevant bioactivities, namely antioxidant, antimicrobial and anti-inflammatory activities [37,38]. Among the most interesting secondary metabolites found in nettle, it is worth mentioning lignans [39,40], as well as phytosterols and pentacyclic triterpenes, such as β-amyrin and oleanolic acid [41]. Additionally, the cortex of nettle stems contains silky and strong bast fibers valued by the biocomposite sector because of their lightweight properties and low C footprint [42]. The aim of the work carried out here is to develop a protein purification protocol so as to define the partitioning of the proteins in different cell compartments and to analyze how SuSy can display different patterns of phosphorylation in relation to different cell compartments. Defining these parameters is of critical importance in order to analyze the function of SuSy during the development of nettle stem plants, especially in relation to bast fibers, which are the most important components from application and economic points of view.

Much of the work undertaken in this manuscript is dependent on the cross-reactivity with a commercial antibody directed against *Arabidopsis* SUS1. According to the manufacturer, the antibody may exhibit reactivity to other species. At present, we have no information on whether the antibody can cross-react with other SuSy isoforms in nettle. The antibody is a polyclonal directed against the entire SUS1 protein. Considering the sequence homology between the various SuSy isoforms in nettle [32], it cannot be excluded that the probe may bind to other isoforms. In any case, the data indicate that the antibody is specific against proteins of expected molecular weight for nettle SuSy.

Nettle SuSy has been identified in several cell fractions, such as cytosol, intracellular membranes and the cell wall. This is not surprising, because a differential distribution of the enzyme has already been described in several case studies, both in somatic and germ cells [11,43,44]. The different distributions of SuSy is a consequence of the different processes in which the protein may be involved. In fact, in the cytosol, SuSy can metabolize sucrose by storing part of its energy in the UDP-glucose molecule. This is very useful in cases of extreme or stressful environmental conditions, where in addition to the production of sugars for glycolysis and respiration, it is also necessary to conserve energy, such as during anaerobic stress [45]. It should, therefore, not be a surprise that even SuSy from nettle stems can be observed in different compartments. While SuSy in the cytosol may have an obvious function, the role of SuSy associated with membranes is still not clear. On the one hand, the SuSy associated with membranes may represent a form of SuSy in transit to other compartments; on the other hand, it may also represent a form of SuSy that is less active or even inactive, and therefore the binding to membranes may represent a form of compartmentalization that is used to make this enzyme inactive. In the literature, however, data exist that support the hypothesis that membrane-associated SuSy is used for the production of *callose* and *cellulose* [7,27,46,47,48]. This assumption is based on experimental evidence and on the fact that the ideal substrate of *callose synthase* and *cellulose synthase* is UDP-glucose, one of the products of sucrose cleavage involving SuSy [45]; in addition, cytosolic invertase might also contribute to *cellulose synthesis* [49]. Evidence that SuSy is associated with the plasma membrane of plant cells reinforces this hypothesis, as does the fact that SuSy is sometimes more present in areas of the cellular plasma membranes in direct contact with consistent deposits of polysaccharides, as in the case of *callose* [23]. The evidence for the presence of SuSy in the cell wall has a different value. Truthfully, we must admit that the amount of experimental data supporting this notion is low, and therefore building a reasonable hypothesis is not easy [11,17]. The evidence of SuSy in the cell wall can be interpreted as the need for a larger amount of SuSy in sites of active polysaccharide synthesis. However, this hypothesis conflicts with the fact that *cellulose synthase* and *callose synthase* require substrates present in the cytoplasmic side of the plasma membrane, which is quite contradictory to the presence of SuSy in the cell wall, i.e., on the opposite side. Further experimental data will certainly be requested to support the hypothesis that the cell wall SuSy may be involved in the synthesis of polysaccharides. To speculate further, we propose that similarly to cell wall invertase, the cell wall SuSy could only split sucrose into the apoplast by releasing monosaccharides that enter the cytoplasm through specific transporters [50].

The release assay of SuSy from the membrane and cell wall fractions was performed for at least two reasons. First, the release conditions could provide information about how SuSy binds to membranes and to the protein–polysaccharide complex of the cell wall. This is also an indication of the relative mobility of the protein within the two matrices. The second finding from this experiment is that the release of SuSy is the prerequisite for purification of the enzyme from different compartments. SuSy was found to be associated with the membrane fraction in a Triton-dependent and Triton-independent manner [12,51]. The fact that SuSy may be released by Triton, but that a large portion of the protein remains in the Triton-resistant membrane fraction, suggests that the enzyme is also associated with detergent-resistant membrane areas; the latter might correspond to the so-called lipid raft [51,52]. These are areas of the membrane often involved in aggregating proteins with specific functions. A typical example is *callose synthesis* [51]; the fact that *callose synthase* might be present in lipid rafts corroborates the presence of SuSy in lipid rafts, or more generally in Triton-resistant lipid areas.

The association of SuSy with the cell wall is mediated by interactions that are mostly weakened by 1.5 M NaCl and partially by CaCl2. This suggests that the protein is hardly movable in the cell wall matrix. In the case of SuSy from tobacco pollen tube samples, the protein was extracted from the cell wall fraction in low ionic strength conditions (0.15 M NaCl) and then almost completely using 1 M NaCl [53]. The type of interaction does not shed light on the role that SuSy may have in the cell wall. However, it is a matter of fact that SuSy is present in the cell wall. The evidence that in some cases truncated forms of SuSy may exist [17] leaves several hypotheses open, including whether it is an inactive form of SuSy or excess SuSy released into the cell wall. An enzymatic analysis after purification could give some information, however to date we have not been able to set up a purification process capable of yielding adequate amounts of the protein.

Understanding the biochemical and physiological mechanisms of an enzyme may also require purification of that protein. Usually the stems of even herbaceous plants cannot be catalogued as simple cases for protein purification. Therefore, in the case of nettle stems, we had to review the protocols described in the literature and adapt one specifically [6,11,54]. We maintained the classic three-step approach by testing various combinations, at the end of which we opted for a simplified version of the purification process that was based on a first step of anion exchange chromatography followed by a step of gel filtration, concluding with a further anion exchange chromatography step with a higher performing column. The control electrophoresis confirmed that the purification process was sufficiently adequate. The entire process was performed on the cytosolic fraction, which was easier to process. We believe that a similar type of process can also be adapted to the membrane-associated SuSy, as well as to the cell wall SuSy. We are encouraged by the evidence that it is possible to release SuSy both from the membranes and from the cell wall.

In the cytosol fraction, SuSy is represented by a protein band of about 80 kDa. After the three purification steps, only a weak accessory band of lower molecular weight was detected. It is difficult to say whether this is an associated polypeptide. In the gel filtration analysis, SuSy was eluted with a native molecular weight of about 350 kDa, which suggests that cytosolic SuSy is a tetramer of the 80 kDa band. These data are in accordance with the literature for SuSy purified from other species [55,56].

The purification protocol has shown how cytosolic SuSy can be purified. The protocol, with due limitations and changes, could also be used to purify SuSy from the membrane and cell wall fraction. However, it should be considered that the amount of protein in the two fractions is not as high as in the cytosol. Our working program involved purifying SuSy from the three compartments and then analyzing it using two-dimensional electrophoresis. As mentioned before, the amount of protein was not always sufficient for a subsequent 2-D electrophoretic analysis. Moreover, the risk of losing some isoforms during the purification process should not be underestimated. We, therefore, preferred a direct two-dimensional analysis of SuSy on the three fractions of the cytosol, membranes and cell wall. The two-dimensional electrophoresis analysis would have allowed a view of the profile in SuSy isoforms, but it would also have allowed verification of the phosphorylation profile of SuSy isoforms.

Among plant post-translational modifications (PTMs), phosphorylation is probably the most studied, with the first studies dating back to the beginning of the twentieth century. As reported by Schulze [57], the phosphorylation status is controlled by the balance between kinase and phosphatase activity. Interestingly, this PTM seems to regulate most of the plant’s metabolic and physiological pathways, including RNA metabolism [58], plant immunity and defense [59], root growth [60] and carbon metabolism [61]. Many studies have also highlighted the involvement of phosphorylation in regulating SuSy activity. It has been shown that phosphorylation regulates protein localization—phosphorylation of the membrane-associated enzyme causes release from the membrane, while dephosphorylation of the soluble enzyme promotes membrane association [62]. In addition to this, in vitro phosphorylation of SuSy selectively activates the cleavage reaction by increasing the affinity of the enzyme for sucrose and UDP, suggesting that phosphorylation may be of regulatory significance [63].

As a first step in studying the phosphorylation profile of nettle SuSy, an in silico approach was used. Results from the NetPhos 3.1 server [33,34] showed that all of the major P sites were in the SuSy domain, and that similarly for the glycosyltransferase domain, serine (S) sites were more phosphorylated than threonine (T), followed by tyrosine (Y). As for the types of kinases, the prevalence of the predicted ones was not specific, while PKA, PKC and CKII were the most common. Interestingly, some of the predicted phosphorylation results agree with in vitro and in vivo studies. For example, as reported in maize, SUS1 S15 is also predicted to be phosphorylated in nettle, which would indicate an alteration of the amino terminal conformation in a way that may stimulate the catalytic activity of SUS and influence membrane association [62]. In addition to this, S170 seemed to be phosphorylated in UdSUS6, as reported in maize [64]. In this study, the authors showed that kinase specificity for S170 was three-fold lower than that for S15, and that phosphorylation of S170 was stimulated by prior phosphorylation at the S15 site. Another interesting site is S11, which is predicted to be phosphorylated in UdSUS3. Similarly, Nakai et al. [65] reported that this amino acid was phosphorylated by a Ca^2+^-dependent protein kinase and that this PTM caused an increase in apparent affinity for sucrose.

To define the isoform profile of SuSy as used in the three main cell fractions (i.e., the cytosol, membranes and cell wall), the proteins of these three fractions were analyzed using two-dimensional electrophoresis. The comparison between the resulting spots showed some significant differences. For example, the cytosolic isoforms of SuSy are concentrated in the most acidic part after 2D electrophoresis. Vice versa, the SuSy isoforms associated with membranes are more concentrated in the cluster of isoforms in the basic parts of the gels. The cell wall SuSy isoforms are distributed in both clusters (acidic and basic), without a clear preference. Separation by 2D electrophoresis followed by staining for phosphoproteins is one of the methods used to highlight the phosphorylation pattern [66]. Analysis of the phosphorylation of the diverse gel isoforms showed that the most acidic SuSy cluster was represented by phosphorylated isoforms, while the most basic cluster was non-phosphorylated. These data suggest that in nettle the phosphorylation of SuSy makes this protein more cytosolic, while the non-phosphorylation allows the association of SuSy with the membrane fraction.

The different phosphorylation patterns of SuSy isoforms may be a way to control the enzymatic activity of the protein, as well as its compartmentalization, such as in sycamore trees [47]. The different phosphorylation patterns of SuSy could also be a control mechanism of the state of oligomerization and the association with actin filaments [67]. The same authors also highlighted how in maize different isoforms of SuSy can exhibit different patterns of phosphorylation, as well as different distributions [43]. In maize, phosphorylation of the amino terminal portion of SuSy determines the association of the protein with different cell compartments [62], as well as in tomato fruit [68]. As already stated, the phosphorylation of SuSy can also affect its enzymatic activity, for example in relation to the synthesis of *callose* [69] or to the increased degradation of sucrose [70]. In other cases, phosphorylation in distinct amino acid residues may be a signal for the proteolytic degradation of the enzyme [64]. In pear, the different phosphorylation patterns of SuSy are associated with distinct stages of development [71].

Although a considerable number of experiments has already been carried out and results have been obtained, we still do not have a precise picture of how phosphorylation can change the distribution and activity of SuSy. In some cases, phosphorylation is mainly associated with cytosolic forms of SuSy, but this is not always exactly the case. Our observations show that even in the stem of nettle (enriched in bast fibers), SuSy can be distributed in different subcompartments, and that this is related to the phosphorylation status of its isoforms. These observations pave the way for further investigations that will allow us to understand how the phosphorylation status of SuSy varies during the development of bast fibers in nettle stems.

## 4. Materials and Methods

### 4.1. Plant Material

*Urtica dioica* L. plants were supplied by the Botanical Garden of the University of Siena. For the reported experiments, the upper third part of the stem was taken, more specifically the internode that formed a kink when gently tilted. This internode corresponds to the so-called snap point previously described in flax [72].

### 4.2. General Protein Extraction from Plant Species

Proteins were extracted from *Pisum sativum*, *Urtica dioica* and *Capsicum annuum* leaves using the following protocol: 1 g of tissue was finely ground using liquid nitrogen and proteins were precipitated by adding 7 mL of cold 10% TCA/acetone and 70 µL of 1 M DTT. Samples were vortexed for 3–5 min, and thus centrifuged (5 min at 4 °C, 15,000× *g*). The resulting pellet was washed twice with 7 mL of cold 80% acetone and dried at room temperature. The pellet was then resuspended in 7 mL of extraction buffer (1% (*w*/*v*) SDS; 0.15 M Tris-HCl (pH 8.8); 0.1 M DTT; 1 mM EDTA; 0.7 M sucrose; 0.06% (*w*/*v*) protease inhibitor mixture) and then incubated for 1 h at room temperature. Samples were centrifuged (10 min at room temperature, 15,000× *g*) and the supernatant was transferred into new tubes. An equal volume of Tris-buffered phenol was added and the samples were vortexed for 3–5 min. The resulting mixture was centrifuged (5 min at room temperature, 15,000× *g*) and the phenolic phase was collected in a new Eppendorf tube, into which 5 mL of 0.1 M ammonium acetate in methanol were added. Samples were, thus, incubated for 30 min at −20 °C and then centrifuged (10 min at 4 °C, 15,000× *g*). The pellet was washed a first time with 5 mL 0.1 M ammonium acetate in methanol; a second wash was performed twice using 5 mL 80% acetone, then the resulting pellet was dried at room temperature and finally resuspended in in 1× Laemmli sample buffer.

### 4.3. Sequential Protein Extraction from Nettle Stem

Proteins were sequentially extracted from nettle stem using the following protocol. Briefly, 2 g of ground tissue was homogenized in 10 mL of Tris-HCl buffer (50 mM, pH 7.5; 0.06% PIC (*w*/*v*, Protease inhibitor mixture); 1 mM EDTA; 1% PVPP; 2 mM DTT), incubated for 30 min at 4 °C under constant agitation and then centrifuged (5 min at 4 °C, 1000× *g*). The pellet was recovered and kept at −80 °C for the next cell wall protein extraction. The supernatant was, thus, centrifuged (1 h at 4 °C, 118,000× *g*). The resulting supernatant represented the cytosolic proteins (HSS) and was resuspended in 1× Laemmli sample buffer; the pellet was resuspended in 200 μL Tris-HCl buffer (50 mM, pH 7.5; 1 mM EDTA; 1% PVPP; 0.1% Triton X-100) and incubated for 15 min on ice. After incubation, the sample was centrifuged (1 h at 4 °C, 118,000× *g*).

### 4.4. Preparation of Soluble (S), Membrane (M) and Cell Wall (CW) Proteins

Soluble proteins (S) were extracted by combining the protocols used by Chabi et al. [73] and Goulas et al. [74]. Briefly, 2 g ground sample was further homogenized for 5 min in 10 mL of cold Tris-HCl buffer (50 mM, pH 7.5, 1 mM EDTA, 1% PVPP, 0.1% BME, plus a mixture of protease and phosphatase inhibitors) and then centrifuged (10 min at 4 °C, 16,000× *g*). The pellet was recovered and stored at −80 °C for cell wall protein (CWP) extraction (see below). The supernatant was further centrifuged for 1 h at 4 °C at 100,000× *g*; the resulting pellet, representing the membrane proteins (M), was resuspended in 1× Laemmli sample buffer and the supernatant was recovered and soluble proteins were precipitated using 60% TCA (*w*/*v*, trichloroacetic acid, 1 h, −20 °C). The pellet was washed twice with 80% cold acetone and dried at room temperature before storage at −20 °C. CWPs were extracted from the CWP pellet as previously described by Feiz et al. [75]. Briefly, the pellet was washed twice with 5 mL sodium acetate buffer (5 mM, pH 4.6) and then incubated with 5 mL 1.5 M NaCl for 30 min on a rocking platform at 4 °C. The solid cell wall residue was then further extracted using two subsequent incubation steps: the first with 5 mL CaCl_2_ buffer (5 mM sodium acetate, 200 mM CaCl_2_, 30 min at 4 °C) and the second with 50 mL LiCl buffer (5 mM sodium acetate, 3 M LiCl, 30 min at 4 °C). The liquid fractions from all CWP extractions were combined and CWPs were precipitated by adding 10% TCA (*w*/*v*). The protein content was determined using reducing agent-compatible (BioRad) and detergent-compatible (Bradford) assays.

### 4.5. Purification of SuSy from Nettle Stem

The cytosolic fraction from nettle stems was obtained according to the protocol described above. The sample was centrifuged at 16,000× *g* (4 °C) for 10 min and was diluted to 1:2 with buffer A (50 mM Tris-HCl pH 7.5; 1 mM EDTA; 1 mM DTT). All chromatography runs were performed using an AKTA Purifier 10 (GE HealthCare). The first chromatographic step was performed on a Hi-Trap Q column (GE HealthCare) equilibrated with buffer A. The protein sample was loaded and fractionated into 0.5 mL fractions with a linear gradient ranging from 0 to 1 M KCl in buffer A (hereafter referred to as buffer B) in 20 column volumes (flow rate of 1 mL/min). A spot test with the anti-SuSy antibody was carried out to determine the positive fractions. In the meantime, the Superdex-200 gel permeation column (Ge HealthCare) was equilibrated according to the manufacturer’s instructions in 50 mL of buffer A. Fractions positive in the spot test were pooled and concentrated using a VivaSpin-2 or VivaSpin-500 instrument. The sample was then loaded onto the Superdex 200 gel permeation chromatography column and fractionated in 0.5 mL fractions (flow rate of 0.8 mL/min). To calibrate the filtration gel column, we used a series of reference marker proteins, namely thyroglobulin, bovine γ-globulin and chicken ovalbumin. A spot test with the anti-SuSy antibody allowed us to determine the positions of immunoreactive fractions. The next step was anion exchange chromatography on a Mini-Q column (GE HealthCare), whereby the sample equilibrated according to the manufacturer’s instructions in buffer A. Fractions positive in the spot test were pooled and eluted using anion exchange chromatography with a Mini-Q column using a linear gradient of 0 to 100% buffer B (flow rate of 1 mL/min), with fractions of 0.5 mL collected. A final spot test with the anti-SuSy antibody was then carried out to pool the immunoreactive fractions. All steps were carried out at room temperature. Absorbance at 280 nm, conductivity and pH were checked simultaneously.

### 4.6. Spot Test

Spot tests to identify the chromatography fractions containing SuSy were performed on nitrocellulose membranes. A volume of 3 µL was spotted for each fraction. The membrane was air-dried then incubated with the blocking agent at a concentration of 5% in Tris-buffered saline at pH 7.5 containing 0.1% Tween 20 (TBS-T) for 1 h. The membrane was then incubated with the anti-SuSy antibody (Agrisera, AS15 2830) and diluted to 1:10,000 in TBS-T for 2 h. After two washes with TBS, the membrane was incubated with horseradish peroxidase-conjugated secondary antibody and diluted to 1:3000 in TBS-T. Two washes with TBS followed, then the signal was detected with ECL Clarity (Bio-Rad, Hercules, CA, USA).

### 4.7. 1-D and 2-D Electrophoresis and Immunoblotting

The 1-D electrophoresis was carried out as described [76] using 10% acrylamide gels. All gels were stained with BioSafe Coomassie (Bio-Rad) using the Precision Protein standard (Bio-Rad). For 2-D electrophoresis, protein samples were dissolved in the ReadyPrep 2-D Cleanup Kit from Bio-Rad. The Immobiline DryStrip gels (11 cm long, Bio-Rad) contained a pH gradient of 4–7. The electrophoretic run in the first dimension was carried out with a first step of 0 to 500 V with 1 h, then kept constant at 500 V for 1 h, followed by a linear gradient of 500 to 4000 V within 2 h, then kept constant at 4000 V for 2 h. A second gradient of 4000 to 8000 V in 2 h was used, followed by being kept constant at 8000 V for a total of 25,000 V/h. Protein samples were directly included in the rehydration buffer. After running, gels were stored at −20 °C or immediately equilibrated in the equilibration buffer (6 M urea; 2% SDS; 0.37 M Tris-HCl pH 8.8; 20% glycerol) containing 130 mM DTT (first wash) and 135 mM iodoacetamide (second wash) for 15 min. Proteins were separated in the second dimension by gel electrophoresis on a Bio-Rad pre-cast XT Criterion Bis-Tris 10% gel. Parallel unstained gels were blotted onto PVDF membranes (Bio-Rad) and probed with antibodies. Immunoblotting was performed using a Turbo Blotter (Bio-Rad). After blotting, membranes were washed in TBS-T and blocked with 5% powdered milk in TBS-T. Primary anti-SuSy antibody (Agrisera, AS15 2830) was diluted 1:10,000 in TBS-T and incubated for 2 h. After several washings in TBS-T, goat anti-rabbit horseradish peroxidase-conjugated secondary antibody (Bio-Rad) was diluted to 1:3000 in TBS-T and incubated for 1 h. Immune reaction was visualized using the ECL Clarity detection reagents (Bio-Rad).

Blots were acquired and analyzed using the Fluor-S Multi-Imager (Bio-Rad) instrument, controlled by the QuantityOne (Bio-Rad) software; the exposure time was 60 s. For the comparison of blots, the PDQuest software (Bio-Rad) was used to align the different spots and to give a relative quantification for each one, highlighting both the qualitative and quantitative differences. The software generates a general reference image through which it aligns the various spots. Finally, the data relative to each single spot was exported and graphed with Microsoft Excel.

### 4.8. Assay of Phosphoproteins on Membranes

The protocol for visualizing phosphorylated proteins on the PVDF membrane was based on the Thermo Fisher Pro-Q Diamond Blot Reagent. Proteins were separated by electrophoresis and then transferred to a PVDF membrane. After protein electroblotting, the membrane was allowed to dry completely. All fixing, washing, staining and de-staining steps were performed by soaking the membrane face down with constant shaking. BSA as the non-phosphorylated protein and ovalbumin as the phosphorylated protein were used as the control. The PVDF membrane was pre-moistened in methanol. Proteins were fixed to the membrane by soaking the membrane in 25 mL of Fix Solution (7% acetic acid; 10% methanol) for 10 min. The membrane was washed by dipping it in about 25 mL of dH2O for about 5 min (four washes). The membrane was stained in 25 mL of the 1000-fold-diluted solution of Pro-Q^®^ Diamond Phosphoprotein Blot for 15 min. The membrane was then de-stained in 30 mL of Destain solution (50 mM sodium acetate; pH 4.0, 20% acetonitrile) three times for 5 min. The staining result was achieved by illuminating the membrane with epi-UV in the Fluor-S apparatus (615 nm long-pass, 10–20 s). To prepare it for probing with antibodies, the membrane was moistened by briefly dipping it in 100% methanol. Then, the membrane was washed in TBS twice for 15 min and incubated in 25 mL of blocking agent at room temperature for 1–2 h (or at 4 °C overnight).

To achieve alignment between the spots identified by the phosphoprotein dye and the spots recognized by the antibody, the membrane was marked in the corners with a pen. In addition, even the pre-stained molecular weight standards were a useful reference point. Individual images (both those obtained with phosphoprotein dye and those obtained by antibody labeling) were then aligned using commercial photo editing software capable of using different transparent layers (no adjustments were made). After alignment, individual images were then cropped to the same size and saved as TIF. Then, the new cropped images were imported into ImageJ by overlaying them as a stack, generating distinct colors—green for phosphoproteins and red for immunoreactive spots.

### 4.9. In Silico Prediction of Phosphorylation Sites

The sequences of the six predicted nettle *SUS* genes were retrieved by blasting the *Arabidopsis SUS* against the available nettle leaf database at Blast4OneKP (http://db.cngb.org/blast4onekp/home). The resulting nettle DNA sequences were translated into protein sequences using the Expasy translating tool (https://web.expasy.org/translate/) with the “standard” genetic code. The Pfam web-based tool and NetPhos 3.1 server [33,34] were used for the functional annotation and the prediction of phosphorylation sites, respectively.
